# Inhibition of phosphodiesterase‐4 in the spinal dorsal horn ameliorates neuropathic pain via cAMP‐cytokine‐Cx43 signaling in mice

**DOI:** 10.1111/cns.13807

**Published:** 2022-02-14

**Authors:** Fang‐fang Zhang, Hao Wang, Yan‐meng Zhou, Hai‐yang Yu, Melanie Zhang, Xian Du, Dong Wang, Feng Zhang, Ying Xu, Ji‐guo Zhang, Han‐Ting Zhang

**Affiliations:** ^1^ Institute of Pharmacology Shandong First Medical University & Shandong Academy of Medical Sciences Tai’an China; ^2^ Department of Neurobiology Northwestern University Feinberg School of Medicine Evanston Illinois USA; ^3^ Department of Pharmaceutical Sciences School of Pharmacy & Pharmaceutical Sciences University at Buffalo the State University of New York Buffalo New York USA; ^4^ Department of Pharmacology School of Pharmacy Qingdao University Qingdao China

**Keywords:** connexin43, neuropathic pain, phosphodiesterase‐4, roflumilast, rolipram

## Abstract

**Background:**

The spinal phosphodiesterase‐4 (PDE4) plays an important role in chronic pain. Inhibition of PDE4, an enzyme catalyzing the hydrolysis of cyclic adenosine monophosphate AMP (cAMP), produces potent antinociceptive activity. However, the antinociceptive mechanism remains largely unknown. Connexin43 (Cx43), a gap junction protein, has been shown to be involved in controlling pain transduction at the spinal level; restoration of Cx43 expression in spinal astrocytes to the normal levels reduces nerve injury‐induced pain. Here, we evaluate the novel mechanisms involving spinal cAMP‐Cx43 signaling by which PDE4 inhibitors produce antinociceptive activity.

**Methods:**

First, we determined the effect of PDE4 inhibitors rolipram and roflumilast on partial sciatic nerve ligation (PSNL)‐induced mechanical hypersensitivity. Next, we observed the role of cAMP‐Cx43 signaling in the effect of PDE4 inhibitors on PSNL‐induced mechanical hypersensitivity.

**Results:**

Single or repeated, intraperitoneal or intrathecal administration of rolipram or roflumilast significantly reduced mechanical hypersensitivity in mice following PSNL. In addition, repeated intrathecal treatment with either of PDE4 inhibitors reduced PSNL‐induced downregulation of cAMP and Cx43, and upregulation of proinflammatory cytokines tumor necrosis factor‐α (TNF‐α) and interleukin‐1β. Furthermore, the antinociceptive effects of PDE4 inhibitors were attenuated by the protein kinase A (PKA) inhibitor H89, TNF‐α, or Cx43 antagonist carbenoxolone. Finally, PSNL‐induced upregulation of PDE4B and PDE4D, especially the PDE4B subtype, was reduced by treatment with either of the PDE4 inhibitors.

**Conclusions:**

The results suggest that the antinociceptive effect of PDE4 inhibitors is contributed by increasing Cx43 expression via cAMP‐PKA‐cytokine signaling in the spinal dorsal horn.

## Graphical abstract

The present study provides not only solid demonstration for the analgesic effects of PDE4 inhibitors but also valued clues on the novel mechanism of cAMP‐PKA‐cytokine signaling whereby PDE4 inhibitors produce analgesic activity. The findings will aid in the development of novel antinociceptive agents.

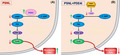

## 1 INTRODUCTION

Neuropathic pain, a chronic nociceptive state that worsens the life quality of 7%–10% general population,^1^ usually results from injury or diseases in the peripheral or central nervous system (CNS). Neuropathic pain is one of the most difficult pain syndromes to manage, it is particularly necessary and important to investigate and develop novel analgesics. Neuropathic pain is contributed by the dysregulation of numerous cellular functions at the spinal cord level.^2^, ^3^ More specifically, activation of glia throughout the neuron system and the subsequent production of proinflammatory molecules from these cells in the spinal dorsal horn are crucial in the mediation of neuropathic pain.^2^ Studies from our laboratory and other groups have demonstrated that proinflammatory cytokines, such as tumor necrosis factor‐α (TNF‐α) and interleukin‐1β (IL‐1β), downregulate the expression of connexin43 (Cx43), a transmembrane protein, in astrocytes.^4^, ^5^, ^6^ Cx43 is highly expressed in spinal astrocytes and plays a pivotal role in the formation of gap junctions^7^, ^8^, ^9^ and, accordingly, it is initially described as a gap junction α‐1 protein (GJA1). Recently, it has been demonstrated that spinal astrocytic Cx43 plays an important role in nociceptive transduction in the neuropathic pain state.^7^, ^10^ Specifically, spinal astrocytic Cx43 is downregulated in the ipsilateral lumbar spinal dorsal horn 7 days after partial sciatic nerve ligation (PSNL) and is restored following treatment of adenovirus vectors expressing Cx43, leading to amelioration of PSNL‐induced mechanical hypersensitivity.^7^ These results suggest that restoration of decreased spinal astrocytic Cx43 can be a potent therapeutic approach to the treatment of neuropathic pain.

Cyclic nucleotide phosphodiesterases (PDEs) consist of a large family of enzymes that catalyze the hydrolysis of the important second messengers cyclic adenosine monophosphate (cAMP) and/or cyclic guanosine monophosphate (cGMP). PDEs play an essential role in regulating the intracellular concentrations of the cyclic nucleotides and in controlling their downstream signal transduction.^11^, ^12^ PDEs are a complex and diverse superfamily of more than 100 different protein products transcribed from 11 distinct but structurally related gene families (PDE1‐11).^13^, ^14^ Among the 11 PDEs, PDE4 has been shown to be the major PDE family responsible for cAMP hydrolysis in nerve and immune cells; and inhibition of PDE4 produces antinociceptive and antiinflammatory effects in the CNS.^14^, ^15^ Since Cx43 is importantly regulated by cAMP signaling,^16^ it is reasonable to believe that PDE4 inhibition may reduce neuropathic pain by modulating the expression of Cx43 in the spinal dorsal horn.

In the current study, we examined the potential role of PDE4 in Cx43 expression and pain‐related behavior using PSNL in mice, a model of peripheral neuropathic pain. In addition, we investigated the possible regulatory role of PDE4‐mediated cAMP‐cytokine signaling in Cx43 expression. The results support a complex interaction between PDE4‐cAMP signaling and Cx43 in the mediation of neuropathic pain.

## 2 MATERIALS AND METHODS

### 2.1 Animals

Male C57BL/6J mice, 5 weeks of age, were purchased from Beijing Vital River Laboratory Animal Technology Co., Ltd. (Beijing, China). Mice were housed in a vivarium in the SPF animal facilities of the Institute of Pharmacology, Shandong First Medical University (Tai'an, China), with constant room temperature (22 ± 2°C) and humidity and a 12‐h light/dark cycle (lights on/off at 8:00 AM/8:00 PM). Animals had access to food and water ad libitum during the experimental period. All experiments utilizing animals were conducted in accordance with the NIH Guide (NIH Publication No. 80–23, revised 1996) for the Care and Use of Laboratory Animals; the procedures were approved by the Laboratory Animals' Ethics Committee of Shandong First Medical University. The animals were treated humanely, and all efforts were made to minimize animals' suffering and the animal numbers. Animal studies are reported in compliance with the ARRIVE guidelines.^17^

### 2.2 Partial sciatic nerve ligation

Under anesthesia with sodium pentobarbital (50 mg/kg, i.p.), mice were subjected to the PSNL surgery, in which a tight ligation of approximately one‐third to one‐half of the diameter of the left sciatic nerve (ipsilateral) was performed with 8–0 silk suture, as described in our previous study.^7^ In sham (control) mice, the sciatic nerve was exposed without ligation using the same procedure. The success rate for PSNL operation was approximately 95%. Mice with PSNL that did not show robust mechanical hypersensitivity (hind paw withdrawal threshold >0.16 g) were excluded from the experiment.

### 2.3 Mouse intrathecal injection

Intrathecal injections were performed following the procedures published elsewhere.^18^, ^19^, ^20^ Briefly, mice were restrained with the experimenter's left hand and the injection was performed with the right hand. The vertebral landmarks for L5 and L6 vertebrae were identified by palpation. Rolipram, roflumilast, or vehicle (30% DMSO in saline) was injected (5 μl in volume) into the subarachnoid space between the L5 and L6 vertebrae via a 27‐gauge needle. The tip of the needle was kept at the injection site for approximately 15 s after completion of the injection to ensure the full delivery of the solution. The entry of the needle was confirmed with the presence of a tail flick.

### 2.4 Intrathecal drug administration and testing schedule

Mice with PSNL were intrathecally injected with rolipram, roflumilast, or vehicle (5 µl) 14 days following surgery; mice with sham surgery were treated similarly with a vehicle as to the naive control. Withdrawal thresholds were measured 0.5, 1, 2, 4, 6, and 24 h post‐injection using the von Frey test; 29 male mice were used in the experiments. For chronic treatment, rolipram, roflumilast, or vehicle was injected intrathecally once daily 7–13 days following PSNL; withdrawal thresholds were measured 24 h after the last intrathecal injection (i.e., 14 days following PSNL). Seventy‐three male mice were used in the experiments. Upon completion of the measurement of withdrawal thresholds, mice were decapitated and the lumbar (L4‐L6) segments of the ipsilateral, spinal dorsal horn were removed. The tissues were immediately frozen in liquid nitrogen and stored at −80°C until use. Expression of PDE4s and Cx43 in the spinal dorsal horn was assessed using Western blotting analysis.

### 2.5 Hind paw sensitivity to mechanical stimulation

The withdrawal threshold (in grams) of the hind paw to mechanical stimulation was determined using von Frey filaments.^21^ Briefly, mice were individually placed in the separate plastic box (625 × 615 × 20 cm) with a metal mesh floor and allowed to acclimate for 45 min. The von Frey filaments (0.008, 0.02, 0.04, 0.07, 0.16, 0.4, 0.6, 1.0, 1.4, and 2.0 g) (North Coast Medical, Inc.) were respectively pressed against the mid‐planter surface of the mouse hind paw. The lowest force that caused responses such as lifting and licking of the hind paw was defined as the withdrawal threshold, which was tested three times in 10‐sec intervals, and the mean withdrawal threshold was calculated. Prior to drug treatment, mice were assessed for baseline withdrawal thresholds and then randomized into different treatment groups. All behavioral tests were performed with the observer blinded to the drug treatment.

### 2.6 Western blotting

The lumbar (L4‐L6) segments of the ipsilateral spinal dorsal horn were collected, immediately frozen in liquid nitrogen, and stored at −80°C until use. The spinal tissues were solubilized in ice‐cold radioimmunoprecipitation assay buffer with protease inhibitors (100 mM Tris‐HCl, pH 7.4, 150 mM NaCl, 1 mM EDTA, 1% Triton ×‐100, 1% sodium deoxycholate, 0.1% sodium dodecyl sulfate, and 1 mM phenylmethylsulfonyl fluoride) and phosphatase inhibitor cocktail 2 (Solarbio, Beijing, China)) with sonication. The lysates were centrifuged at 13,000 × *g* at 4°C for 10 min and the supernatant was added to Laemli's buffer and boiled for 5 min. Equal amounts of protein were separated by 7.5% or 10% sodium dodecyl sulfate‐polyacrylamide gel electrophoresis and blotted onto nitrocellulose membranes. Non‐specific binding was reduced with blocking buffer (20% skim milk in Tris‐buffered saline with Tween 20), and the membranes were subsequently incubated with purified horseradish peroxidase (HRP)‐conjugated monoclonal antibodies against PDE4A, PDE4B, PDE4C, or PDE4D (all in 1:500; Abcam Biochemicals), polyclonal antibodies against Cx43 (1:1000; Abcam Biochemicals), or monoclonal antibodies against β‐actin (1:10 000; Sigma Chemical Co.) at 4°C overnight. After washing, the membranes were incubated with HRP‐conjugated secondary antibodies (Zsbio Commerce Store) at 25°C for 2 h. Membranes were then rinsed and incubated with ECL luminescence reagent (Absin) before exposure using Image‐Pro Plus software version 6.0 (Media Cybernetics Corp).

### 2.7 Enzyme‐linked immunoassay assay

The spinal cord tissues were homogenized with ice‐cold phosphate‐buffered solution containing 1% phenyl methane sulfonyl fluoride. Lysates were repeatedly thawed and refrozen three times and the supernatants were collected after centrifugation at 5000 ×*g* for 10 min. The contents of TNF‐α, IL‐1β, IL‐6, cAMP, and cGMP in the spinal cord tissues were determined using ELISA kits (Elabscience) following the instructions of the manufacturer; each sample was assessed in duplicates. The colorimetric reaction was conducted and the absorbance at 450 nm was recorded using a multifunctional microplate reader (TECAN). Protein concentrations of samples were determined using enhanced BCA protein assay kits (Solarbio) according to the manufacturer's instructions.

### 2.8 Statistical analysis

**FIGURE 1:**
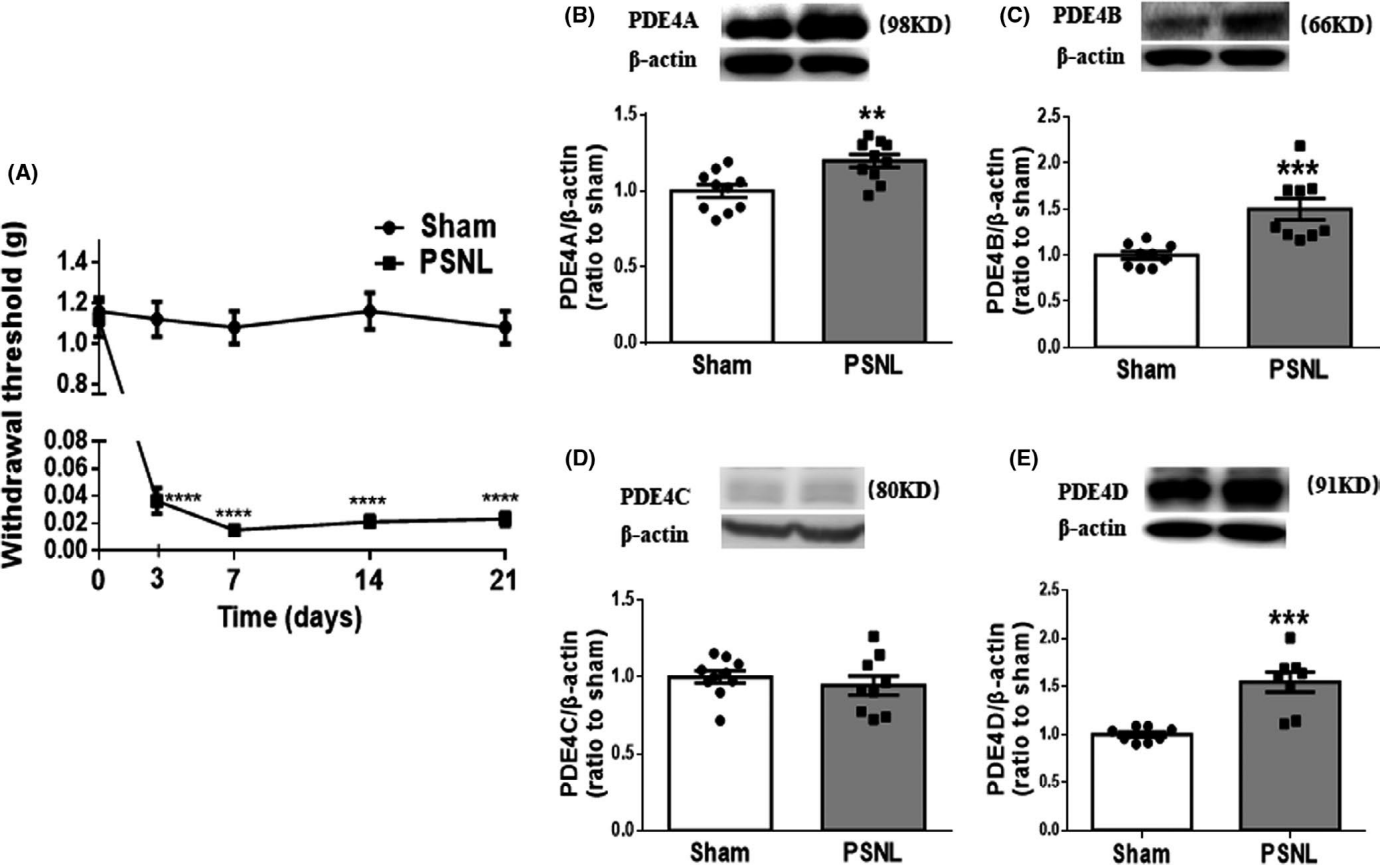
Partial sciatic nerve ligation (PSNL) produced mechanical hypersensitivity and increased expression of PDE4 subtypes in the spinal dorsal horn in mice. (A) Ipsilateral hind paw withdrawal thresholds (g) over time measured with von Frey filaments. Mice were tested before (0 day), and 3, 7, 14, and 21 days after PSNL or sham surgery. (B‐E) The expression of PDE4A‐D in the ipsilateral spinal dorsal horn of mice subjected to PSNL or sham, quantified by Western blotting analysis 14 days after surgery. The optic densities of PDE4A‐D were normalized to those of sham controls. Data shown are means ± SEM. ^**^ *p* < 0.01, ^***^ *p* < 0.001, ^****^ *p* < 0.0001 vs. corresponding sham; *n* = 10 (A) or 8–10 per group (B‐E)

**FIGURE 2:**
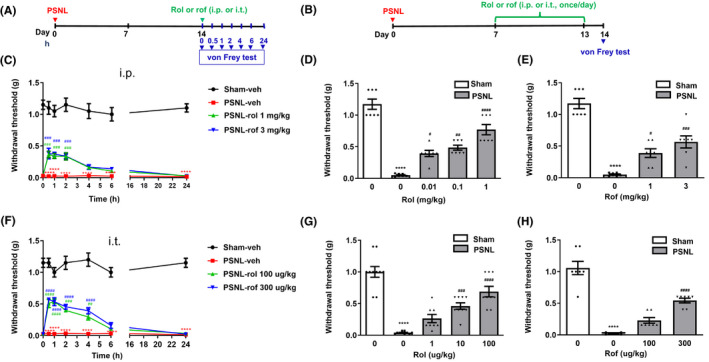
Intraperitoneal or intrathecal treatment with PDE4 inhibitors ameliorated mechanical hypersensitivity induced by PSNL in mice. (A) Schedule of acute PDE4 inhibitor administration and testing. Rolipram (rol), roflumilast (rof), or vehicle (veh) was intraperitoneal (i.p.) or intrathecally (i.t.) injected 14 days after PSNL; hind paw withdrawal thresholds (g) were determined using the von Frey test at 0, 0.5, 1, 2, 4, 6, and 24 h after the injection. (B) Schedule of repeated injections of PDE4 inhibitors. Rolipram, roflumilast, or vehicle was injected (i.p. or i.t.) 7 times (Days 7, 8, 9, 10, 11, 12, and 13 after PSNL surgery). Mice were tested for withdrawal thresholds using the von Frey test on Day 14. (C) Changes in hind paw withdrawal thresholds overtime after acute treatment (i.p.) with rolipram (1 mg/kg), roflumilast (3 mg/kg), or vehicle. (D, E) Effects of repeated treatment (i.p.) with rolipram (0.01, 0.1, or 1 mg/kg) (D) or roflumilast (1 or 3 mg/kg) (E) on hind paw withdrawal thresholds (g) 14 days following PSNL or sham surgery. (F) Changes in withdrawal thresholds (g) following single, intrathecal injections of rolipram (100 µg/kg), roflumilast (300 µg/kg), or vehicle at 0, 0.5, 1, 2, 4, 6, and 24 h after the injection. (G, H) Effects of repeated intrathecal treatment with rolipram (1, 10, or 100 µg/kg) (G) or roflumilast (100 or 300 µg/kg) (H) on hind paw withdrawal thresholds (g) measured by the von Frey test 14 days following PSNL surgery. Data shown are means ± SEM. ^****^ *p* < 0.0001 vs. sham with vehicle; ^#^ *p* < 0.05, ^##^ *p *< 0.01, ^###^ *p* < 0.001, ^####^ *p* < 0.0001 vs. PSNL with vehicle; *n* = 7–10 per group

**FIGURE 3:**
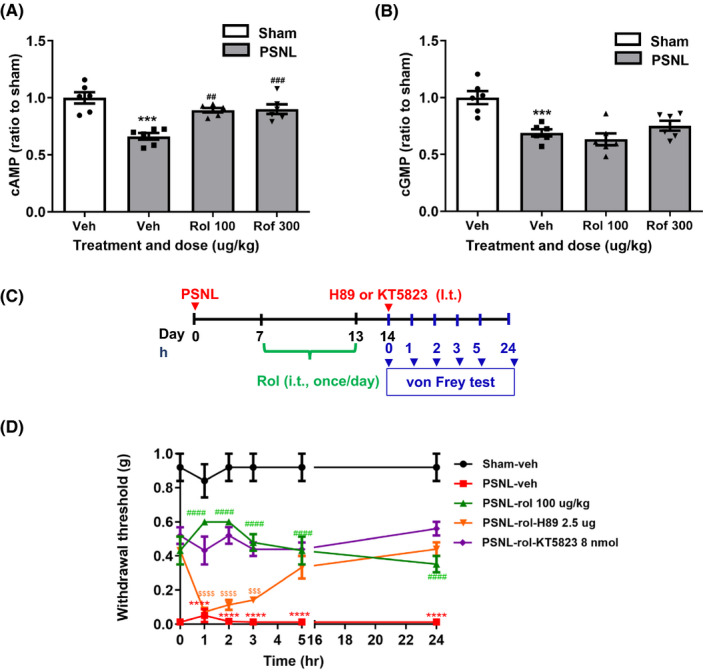
The role of rolipram or roflumilast on cAMP‐PKA signaling in the spinal dorsal horn. (A) Effect of repeated intrathecal treatment with vehicle (veh), rolipram (rol, 100 µg/kg), or roflumilast (rof, 300 µg/kg) on cAMP (A) and cGMP (B) levels in the spinal dorsal horn of PSNL mice. Rolipram (100 µg/kg), roflumilast (300 µg/kg), or vehicle was intrathecally injected once a day for 7 days following the schedule described in Figure 2B. Mice were sacrificed, and the spinal dorsal horn was collected 14 days after PSNL surgery for the determination of cAMP and cGMP levels using ELISA kits. (C) Schedule of drug injections and testing. Rolipram or vehicle was intrathecally injected once a day for 7 days (i.e., 7–13 days after PSNL); H89 or KT5823 was intrathecally injected on Day 14. The hind paw withdrawal thresholds were determined using the von Frey test at 0, 1, 2, 3, 5, and 24 h after the injection. (D) Effects of rolipram (100 µg/kg) on hind paw withdrawal thresholds (g) over time with or without the intrathecal injection of H89 (2.5 µg/5 µl) or KT5823 (8 nmol) in PSNL mice. Data shown are means ± SEM. ^**^ *p* < 0.01, ^***^ *p* < 0.001, ^****^ *p* < 0.0001 vs. sham with vehicle; ^##^ *p* < 0.01, ^###^ *p* < 0.001, ^####^ *p* < 0.0001 vs. PSNL with vehicle; ^$$$^ *p* < 0.001, ^$$$$^ *p* < 0.0001 vs. PSNL with rolipram; *n* = 5–6 per group

**FIGURE 4:**
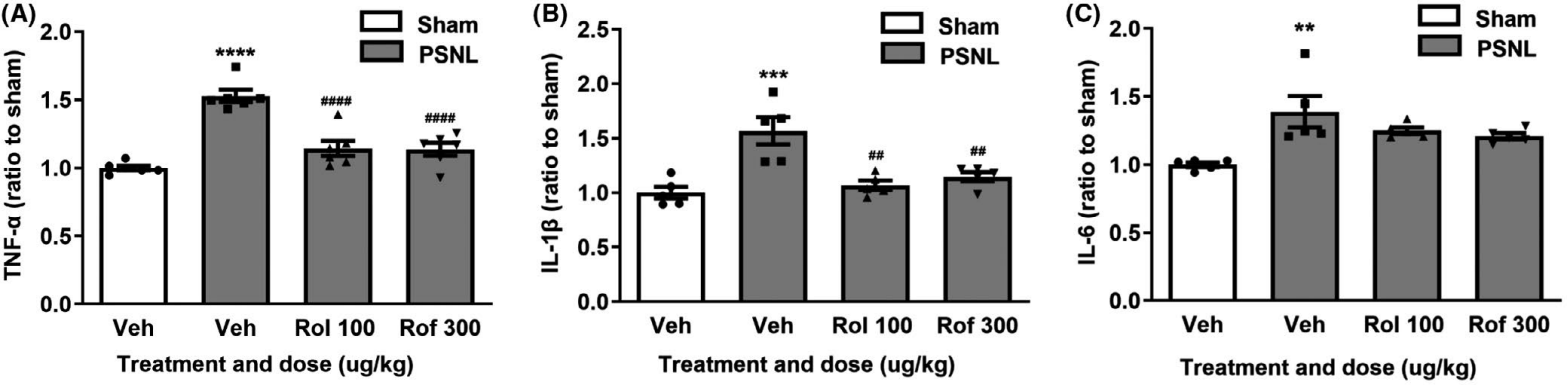
Effects of rolipram or roflumilast on levels of TNF‐α (A), IL‐1β (B), and IL‐6 (C) in the spinal dorsal horn of PSNL mice. Rolipram (rol, 100 µg/kg), roflumilast (rof, 300 µg/kg), or vehicle (veh) was intrathecally injected once a day for 7 days following the schedule described in Figure 2B. Mice were sacrificed, and the spinal dorsal horn was collected 14 days after PSNL surgery for determination of TNF‐α (A), IL‐1β (B), and IL‐6 (C) levels using ELISA kits. Data shown are means ± SEM. ^**^ *p* < 0.01, ^***^ *p* < 0.001, ^****^ *p* < 0.0001 vs. sham with vehicle; ^##^ *p* < 0.01, ^####^ *p* < 0.0001 vs. PSNL with vehicle; *n* = 5–6 per group

**FIGURE 5:**
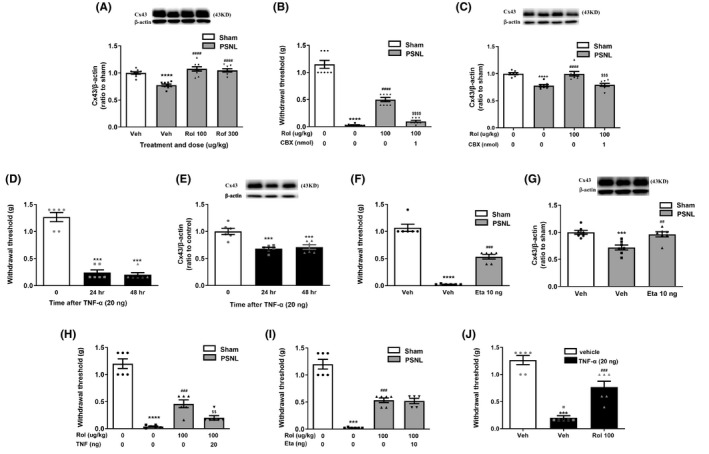
Effects of PDE4 inhibitors on cytokine‐Cx43 signaling in the spinal dorsal horn of PSNL mice. (A) Expression of Cx43 by Western blotting in the spinal dorsal horn of PSNL mice treated with vehicle (veh), rolipram (rol, 100 µg/kg), or roflumilast (rof, 300 µg/kg). Rolipram, roflumilast, or vehicle was intrathecally injected once a day for 7 days, as identified in Figure 2B. (B, C) Rolipram (rol, 100 µg/kg) was intrathecal injected 7 times (7, 8, 9, 10, 11, 12, and 13 days after PSNL surgery) and CBX (1 nmol) was intrathecally injected once on Day 13. Hind paw withdrawal thresholds (g) (B) and Cx43 expression in the spinal dorsal horn (C) were measured using the von Frey test and western blot 14 days following PSNL surgery. (D, E) TNF‐α decreased hind paw withdrawal thresholds (D) and Cx43 expression in the spinal dorsal horn (E) assessed 24 and 48 h after the last intrathecal injection. TNF‐α (20 ng) was intrathecally injected, and hind paw withdrawal thresholds were measured 24 and 48 h after the injection (D and E). (F, G) The effect of etanercept on hind paw withdrawal thresholds (F) and Cx43 expression (G) in PSNL mice. Etanercept (Eta, 10 ng) was intrathecally injected 7 times (7, 8, 9, 10, 11, 12, and 13 days after PSNL surgery), and hind paw withdrawal thresholds were measured on Day 14 (F and G). The spinal dorsal horn was collected for Western blotting for Cx43 expression immediately after the behavioral test. (H) TNF‐α blocked the antinociceptive effect of rolipram. Rolipram (rol, 100 µg/kg) was intrathecally injected 7 times (7, 8, 9, 10, 11, 12, and 13 days after PSNL surgery), and TNF‐α was singly and intrathecally injected on the 13th day. (I) Etanercept did not affect the antinociceptive effect of rolipram. Rolipram (100 µg/kg) was co‐injected intrathecally with etanercept (Eta, 10 ng) 7 times (7, 8, 9, 10, 11, 12, and 13 days after PSNL surgery). For both (H) and (I), mice were tested for hind paw withdrawal thresholds (g) 14 days post‐PSNL after the last intrathecal injections of rolipram with TNF‐α (20 ng) or Eta using the von‐Frey test. (J) Rolipram blocked the nociceptive effect of TNF‐α. Rolipram (100 µg/kg/day) was intrathecally injected for 5 days and TNF‐α (20 ng) injected 2 days before the hind paw withdrawal thresholds were measured using the von Frey test. Data shown are means ± SEM. ^**^ *p* < 0.01, ^***^ *p* < 0.001, ^****^ *p* < 0.0001, vs. vehicle or sham with vehicle; ^##^ *p* < 0.01, ^###^ *p* < 0.001, ^###^ *p* < 0.001 vs. PSNL with vehicle; n = 3 (C) or 9 per group (A). ^$$$^ *p* < 0.001, ^$$$$^ *p* < 0.0001 vs. PSNL with rolipram. *n* = 5–9 per group

**FIGURE 6:**
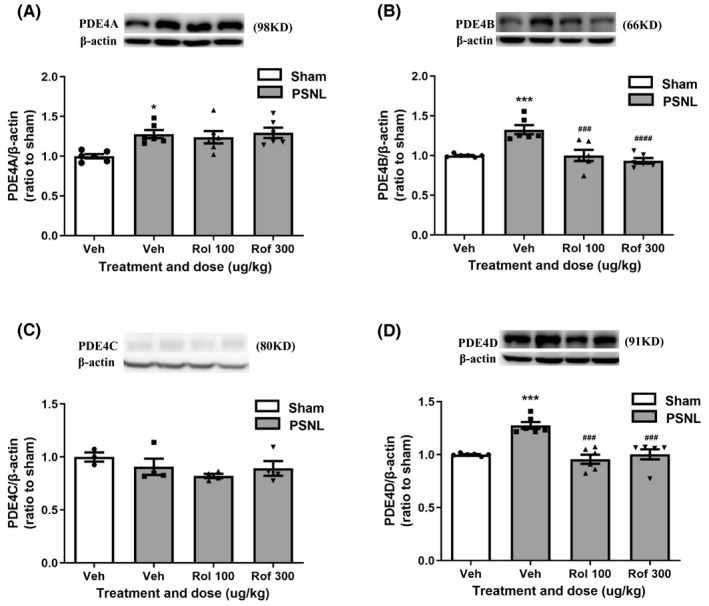
Effects of PDE4 inhibitors on expression of PDE4 subtypes in the spinal dorsal horn in PSNL mice. (A–D) Effect of repeated intrathecal injections of rolipram or roflumilast on expression of PDE4A (A), PDE4B (B), PDE4C (C), and PDE4D (D) in the spinal dorsal horn using Western blotting. Rolipram (rol, 100 µg/kg) or roflumilast (rof, 300 µg/kg) was given following the schedule in Figure 2B. Data shown are means ± SEM. ^*^ *p* < 0.05, ^***^ *p* < 0.001, vs. sham with vehicle; ^###^ *p* < 0.001, ^####^ *p* < 0.0001 vs. PSNL with vehicle; n = 6 per group

All quantitative data were expressed as the means ± standard errors of the means (SEM). Comparisons of PDE4 protein levels after PSNL surgery were performed using student's *t*‐test (for Figure 1B‐E). Comparisons between treatment groups and the corresponding control groups for mechanical hypersensitivity after PSNL (for Figure 2D‐E, G‐H and Figure 5B, D, F, H‐J), levels of cAMP, cGMP, TNF‐α, IL‐1β, IL‐6, Cx43, and PDE4s in the spinal dorsal horn after drug treatment (for Figure 3A‐B, Figure 4A‐C, Figure 5A, C, E, G and Figure 6A‐D were performed using a one‐way analysis of variance (ANOVA) with a pairwise comparison by the Tukey–Kramer method. Possible interaction between PDE4 inhibitor treatment and withdrawal thresholds following PSNL (for Figures 1, 2, Figure 2C and F) and between protein kinase A (PKA) or protein kinase G (PKG) inhibitor treatment and withdrawal thresholds following PSNL (Figure 3D) were analyzed by two‐way ANOVA, followed by the Tukey–Kramer method for post hoc comparisons. Differences were considered to be significant when the *p*‐value was less than 0.05.

## 3 RESULTS

### 3.1 PSNL‐induced mechanical hypersensitivity and expression of PDE4 isoforms in the spinal dorsal horn

We used PSNL to establish the pain model. The withdrawal thresholds of the ipsilateral hind paw dropped from approximately 1.0 g to nearly 0 g in C57B6/LJ mice beginning 3 days after PSNL surgery; this mechanical hypersensitivity persisted up to 21 days following surgery (*F* (4, 36) = 35.37, *p *< 0.0001; Figure 1A). By contrast, no significant changes in withdrawal thresholds were observed following the sham operation.

To determine the involvement of PDE4 subtypes in mechanical hypersensitivity, we examined the expression levels of PDE4A, PDE4B, PDE4C, and PDE4D in the spinal dorsal horn 14 days post‐PSNL using Western blotting. The expression of the PDE4A, PDE4B, and PDE4D in the ipsilateral dorsal horn was significantly increased after PSNL (*t *= 3.362, df = 18 for PDE4A; *t *= 4.077, df = 16 for PDE4B; and *t *= 5.058, df = 14 for PDE4D, *p* < 0.01), while the expression of PDE4C was overall very low and not changed, compared to corresponding sham controls (Figure 1B, C, D, E), suggesting that PDE4A, B, and D may mediate PSNL‐induced mechanical hypersensitivity.

### 3.2 PSNL‐induced mechanical hypersensitivity was attenuated by single or repeated treatment with rolipram or roflumilast

To determine the role of PDE4 in neuropathic pain, we examined the effect of rolipram, a prototypic PDE4 inhibitor, and roflumilast, the first PDE4 inhibitor approved by the Food and Drug Administration for clinic use,^22^ on PSNL‐induced mechanical hypersensitivity following the schedule shown in Figure 2A. Single intraperitoneal (i.p.) treatment with rolipram (1 mg/kg) or roflumilast (3 mg/kg) or intrathecal (i.t.) treatment with rolipram (100 µg/kg) or roflumilast (300 µg/kg) significantly attenuated PSNL‐induced mechanical hypersensitivity (Figure 2C). Specifically, the antinociceptive effects reached the peak 30–60 min after the i.p. injection of PDE4 inhibitors and started to go down 2 h after drug administration and, at 4 h post‐injection, decreased to the levels that were not significantly different from the vehicle control in PSNL mice (Figure 2C). Similarly, rolipram (0.01, 0.1, or 1 mg/kg) or roflumilast (1 or 3 mg/kg) administered repeatedly (i.p., once a day for 7 days, Figure 2B) also increased hind paw withdrawal thresholds of PSNL mice in a dose‐dependent manner relative to PSNL plus vehicle (*F* (4, 30) =51.89, *p* < 0.05 for rolipram; *F* (3, 24) =43.22, *p* < 0.001 for roflumilast; Figure 2D, E), suggesting peripheral administration of PDE4 inhibitors, regardless of acute or chronic treatment, attenuates neuropathic pain. Furthermore, the antinociceptive effects reached the peak in 30–60 min after the i.t. injection, which the effects were reduced and, 6 h post‐treatment, to levels that were no longer significantly different from the vehicle alone in PSNL mice (Figure 2A, F). By contrast, in sham‐operated mice, the PDE4 inhibitors did not alter withdrawal thresholds (data not shown). Similar to the single treatment, repeated i.t. injections (once a day for 7 days, Figure 2B) of rolipram (1, 10, or 100 µg/kg) or roflumilast (100 or 300 µg/kg) increased hind paw withdrawal thresholds of PSNL mice in a dose‐dependent manner relative to PSNL plus vehicle (*F* (4, 41) =35.79 for rolipram; *F* (3, 24) =57.03 for roflumilast; *p* < 0.0001 for both drugs; Figure 2G, H). By contrast, the PDE4 inhibitors did not alter withdrawal thresholds in sham‐operated mice (data not shown). These results suggest that PDE4 in the spinal cord is involved in the antinociceptive effect of the PDE4 inhibitors, regardless of acute or repeated administration.

### 3.3 PDE4 inhibitors ameliorated PSNL‐induced mechanical hypersensitivity via cAMP‐PKA‐cytokines signaling

To determine the role of cAMP signaling in the effect of PDE4 inhibitors on PSNL‐induced mechanical hypersensitivity, we examined the levels of cAMP and cGMP in the spinal dorsal horn of PSNL mice treated with rolipram or roflumilast using enzyme‐linked immunoassay (ELISA) assay. The levels of both cAMP and cGMP were significantly decreased in the spinal dorsal horn of PSNL mice (*p* < 0.001 for cAMP and cGMP; Figure 3A, B). PSNL‐induced decreases in cAMP were reversed by repeated administration (i.t.) of rolipram (100 µg/kg) or roflumilast (300 µg/kg) (*F* (3, 20) =14.95 for cAMP; *p* < 0.01, respectively; Figure 3A). By contrast, neither rolipram nor roflumilast affected PSNL‐induced reduction of cGMP levels in the spinal dorsal horn (Figure 3B).

To further verify the role of cAMP signaling in the antinociceptive effect of PDE4 inhibitors, we examined the effect of H89, a PKA inhibitor, and KT5823, a PKG inhibitor, on the antinociceptive activity of rolipram as the prototypical, selective PDE4 inhibitor in PSNL mice (Figure 3C). As shown in Figure 3D, repeated intrathecal injections of rolipram at 100 µg/kg significantly increased the withdrawal thresholds in PSNL mice; this was reversed by a single, intrathecal injection of H89 (2.5 µg/5 µl), but unaltered by KT5823 (8 nmol). The results strongly support that cAMP‐PKA signaling plays a major role in PDE4‐mediated mechanical hypersensitivity.

Since activation of cAMP‐PKA signaling decreases proinflammatory cytokines such as TNF‐α, IL‐1β, and IL‐6,^23^, ^24^, ^25^ which are associated with neuropathic pain,^26^ we investigated whether the cytokines in the spinal dorsal horn were involved in the antinociceptive effects of the PDE4 inhibitors in PSNL mice. The levels of TNF‐α, IL‐1β, and IL‐6 were significantly increased in the spinal dorsal horn of PSNL mice (*p* < 0.0001 for TNF‐α; *p* < 0.01 for IL‐1β and IL‐6; Figure 4A, B, C). PSNL‐induced increases in the cytokines were reversed by intrathecal administration of rolipram (100 µg/kg) or roflumilast (300 µg/kg) (*F* (3, 20) =26.83 for TNF‐α, *p* < 0.0001 or *F* (3, 16) =11.94 for IL‐1β, *p* < 0.01; Figure 4A, B), except for PSNL‐induced upregulation of IL‐6, which was not altered by the PDE4 inhibitors (Figure 4C).

### 3.4 PDE4 inhibitors ameliorated PSNL‐induced mechanical hypersensitivity via cytokines‐Cx43 signaling

Since Cx43 is highly expressed in astrocytes in the spinal dorsal horn and is importantly involved in PSNL‐induced mechanical hypersensitivity,^7^ we examined the effect of PDE4 inhibitors on Cx43 expression in the spinal dorsal horn of PSNL mice. As demonstrated by Western blotting, expression of Cx43 in the spinal dorsal horn was significantly decreased 14 days after PSNL (*p* < 0.01; Figure 5A). This was reversed by repeated, intrathecal administration of rolipram (100 µg/kg) or roflumilast (300 µg/kg) (*F* (3, 31) =23.07, *p* < 0.0001).

To determine whether PDE4 inhibitors attenuated neuropathic pain via Cx43 in the spinal cord, we examined the effect of CBX, a Cx43 inhibitor, on antinociceptive activity of rolipram in PSNL mice using the von‐Frey test. As shown in Figure 5B, C, PSNL‐induced decrease in withdrawal thresholds and Cx43 expression in the spinal dorsal horn were significantly attenuated by repeated administration of rolipram (100 µg/kg, i.t.; *p* < 0.0001 for threshold, Figure 5B; *p* < 0.01 for Cx43, Figure 5C); these were completely blocked by CBX at 1 nmol (*F* (3, 28) =147.1, *p* < 0.0001 and *F* (3, 27) =18.28, *p* < 0.001 for threshold and Cx43, respectively), a dose that reduces expression of Cx43 in the spinal cord.^7^ The results suggest that the antinociceptive effect of PDE4 inhibitors was mediated by Cx43 in the spinal dorsal horn.

To determine the role of proinflammatory cytokines in mechanical hypersensitivity and Cx43 expression, we examined the effect of TNF‐α on hind paw withdrawal thresholds using the von Frey test. Twenty‐four and 48 h after the intrathecal injections of TNF‐α, withdrawal thresholds were significantly decreased (*F* (2, 15) =97.30, *p* < 0.001; Figure 5D) and expression of Cx43 was also reduced in the spinal dorsal horn (*F* (2, 15) =10.65, *p* < 0.001; Figure 5E). Consistent with these, PSNL‐induced mechanical hypersensitivity and decreases in Cx43 expression were blocked by repeated intrathecal injections of etanercept (10 ng), a TNF‐α inhibitor (*F* (2, 15) =131.0, *p* < 0.001 for threshold and *F* (2, 18) =12.50, *p* < 0.01 for Cx43; Figure 5F, G).

To determine whether TNF‐α was directly involved in the antinociceptive effect of PDE4 inhibitors, we examined the effect of TNF‐α or its inhibitor etanercept in combination with rolipram on mechanical hypersensitivity using the von Frey test. As shown in Figure 5H, I, J, rolipram alone attenuated PSNL‐induced mechanical hypersensitivity (*p* < 0.001); this was blocked by TNF‐α (*F* (3, 20) =70.82, *p* < 0.01; Figure 5H), but not altered by etanercept (Figure 5I) in PSNL mice. Interestingly, rolipram also attenuated TNF‐α‐induced mechanical hypersensitivity (*F* (2, 15) =41.70, *p* < 0.001; Figure 5J). These data suggest that TNF‐α in the spinal dorsal horn is involved in the antinociceptive effect of PDE4 inhibitors.

### 3.5 Effect of PDE4 inhibitors on expression of PDE4 subtypes in the spinal dorsal horn in PSNL mice

To identify the PDE4 subtypes mediated in the mechanical hypersensitivity, we examined the effect of rolipram and roflumilast on expression of specific PDE4s in the spinal dorsal horn in PSNL mice. All PDE4s but PDE4C were increased in the spinal dorsal horn 14 days following PSNL (*p* < 0.0001 for PDE4A, *p* < 0.01 for PDE4B and *p* < 0.001 for PDE4D; Figure 6A, B, C, D). PSNL‐induced upregulation of PDE4B and PDE4D was significantly decreased by repeatedly intrathecal administration of rolipram (*F* (3, 20) =23.25, 100 µg/kg; *p* < 0.01 for PDE4B and *F* (3, 20) =16.53, *p* < 0.001 for PDE4D) or roflumilast (300 µg/kg; *F* (3, 20) =23.25, *p* < 0.01 for PDE4B and *F* (3, 20) =16.53, *p* < 0.001 for PDE4D; Figure 6B, D). By contrast, neither rolipram nor roflumilast significantly altered expression of PDE4A or PDE4C; PDE4A and PDE4C in PSNL mice following treatment with the PDE4 inhibitors (Figure 6A, C).

## 4 DISCUSSION

In the present study, we determined the role of PDE4 in regulating neuropathic pain and its intracellular signaling mechanisms. Our results demonstrated for the first time that PSNL‐induced mechanical hypersensitivity was attenuated by single or repeated treatment (i.p. or i.t.) with rolipram, a prototypical, selective PDE4 inhibitor, or roflumilast, the second generation of PDE4 inhibitors. The antinociceptive effects of the PDE4 inhibitors were blocked or attenuated by the PKA inhibitor H89, TNF‐α, or the Cx43 antagonist CBX, and were related to increases in levels of cAMP and expression of Cx43 and decrease in inflammatory cytokines, including TNF‐α and IL‐1β in the spinal cord. Together, these novel results suggest that the antinociceptive effect of PDE4 inhibitors is mediated by PDE4‐Cx43 signaling.

As PDE4 is the major enzyme hydrolyzing cAMP in cells of the CNS,^13^, ^14^, ^22^, ^27^ inhibition of PDE4 produces beneficial effects in a variety of CNS disorders, including depression,^12^, ^27^ anxiety,^22^ memory loss,^28^, ^29^, ^30^ and alcoholism.^31^, ^32^ Here, we demonstrated that peripheral or intrathecal injections of either rolipram or roflumilast ameliorated PSNL‐induced neuropathic pain in mice. In addition, repeated treatment with the PDE4 inhibitors reversed PSNL‐induced increases in PDE4 expression in the spinal dorsal horn. The results are supported by the finding that intrathecal administration of rolipram ameliorates bone cancer pain.^15^ Consistent with the antinociceptive effect of PDE4 inhibition, knockdown of PDE4B by the intrathecal injection of PDE4B siRNAs attenuates L5 nerve ligation‐induced nociceptive activity.^33^ These data suggest that PDE4 is involved in the regulation of neuropathic pain; PDE4B may be one of the PDE4 isoforms mediating the antinociceptive effect of PDE4 inhibitors.

Rolipram down‐regulates the expression and function of PDE4s.^34^ Roflumilast also inhibits all the PDE4 subtypes to a similar extent but is less potent relative to rolipram.^35^ However, although rolipram and roflumilast have neuroprotective effects, it is not clear if one or more PDE4 subtypes in the brain are involved. Here, we examined the expression of PDE4 isoforms in the spinal dorsal horn of PSNL mice treated with the PDE4 inhibitors. It was interesting that PSNL increased the expression of all but the PDE4C isoform, in particular PDE4B, which was doubled following PSNL. Consistent with this, PDE4B and PDE4D are enriched while PDE4A and PDE4C are very limitedly expressed in the spinal cord.^33^, ^36^ Repeated administration of the PDE4 inhibitors reduced PSNL‐induced upregulation of PDE4B and PDE4D, but not PDE4A, to the levels of naive controls. Given that only knockdown of PDE4B, rather than PDE4A or PDE4D, attenuates neuropathic pain induced by L5 spinal nerve ligation,^33^ it is reasonable to believe that PDE4B is the major PDE4 subtype that mediates neuropathic pain. Nevertheless, the role of PDE4D cannot be excluded given that PDE4D is relatively highly expressed in the spinal cord^36^ and that the PSNL‐induced increase in PDE4D was responsive to PDE4 inhibitor treatment. Further studies will be needed to verify this.

As observed in the current study, PSNL‐induced decreases in cAMP, but not cGMP, were reversed by treatment with either rolipram or roflumilast. In addition, the antinociceptive effect of rolipram was significantly attenuated by H89, a PKA inhibitor, but not by KT5823, a PKG inhibitor. These results strongly support that PDE4 mediates neuropathic pain via cAMP‐PKA signaling in the spinal dorsal horn. It is also supported by other's study that inhibition of the cAMP‐PKA signaling pathway reduces chronic pain in bone cancer.^37^ This is consistent with the effect of opioids, which decrease cAMP and produce analgesia.^38^ However, other studies have also shown the opposite effect, that is, activation of cAMP signaling ameliorates chronic pain caused by L5 spinal nerve ligation,^33^ which is consistent with our result in the present study. While it is not clear what causes the discrepancy, the cAMP levels in different nociceptive models may account at least partially for the different outputs of nociception or antinociception.

It is known that proinflammatory cytokines such as TNF‐α, IL‐1β, and IL‐6 are released in the spinal dorsal horn in response to a variety of pain stimuli.^3^, ^39^, ^40^, ^41^, ^42^ This was demonstrated in the present study in PSNL mice, which showed increases in all three cytokines. Interestingly, PSNL‐induced increases in the cytokines, in particular TNF‐α and IL‐1β, were reversed by inhibition of PDE4 with either rolipram or roflumilast. The results are supported by recent studies showing that rolipram reduces bone cancer pain in rats via its antiinflammatory activity.^15^ Since inhibition of PDE4 decreases the levels of cytokines and produces antinociceptive effects,^14^, ^15^ it is considered that proinflammatory cytokines contribute to PDE4‐mediated pain. Of note, PSNL‐induced upregulation of IL‐6 was not changed by the PDE4 inhibitors, indicating that the response of proinflammatory cytokines to PDE4 inhibition may differ in different disease models.

Cx43 is an important mediator in neuropathic pain and astrocytic function.^10^, ^43^ It has been shown that astrocytic Cx43 expression is significantly decreased in the spinal dorsal horn of PSNL mice.^7^ Thus, we hypothesized that Cx43 is a player in PDE4‐mediated neuropathic pain. This was demonstrated by our result that treatment with the PDE4 inhibitors reversed PSNL‐induced downregulation of Cx43 in the spinal dorsal horn. It was further supported by the result that CBX, a Cx43 antagonist and a gap junction inhibitor, blocked rolipram‐induced antinociceptive effects in PSNL mice. These results suggest that Cx43 is importantly involved in the antinociceptive activity of PDE4 inhibitors.

In addition, a number of in vitro and in vivo studies have demonstrated that proinflammatory cytokines such as TNF‐α decrease Cx43 expression in astrocytes.^5^, ^7^ Consistent with this, we also demonstrated that TNF‐α mimicked the ability of PSNL to produce mechanical hypersensitivity and decrease Cx43 expression in the spinal dorsal horn, both of which were blocked by the TNF‐α inhibitor etanercept. Pretreatment with rolipram or roflumilast reversed PSNL‐induced increases in TNF‐α, and the antinociceptive effect of rolipram was blocked by TNF‐α, but not affected by etanercept. Given that TNF‐α‐induced nociception was blocked by rolipram, PDE4 appears to interact with TNF‐α in the regulation of neuropathic pain. These results suggest that PDE4 inhibition‐induced antinociception is mediated by TNF‐α and subsequently Cx43 in the spinal dorsal horn.

It is known that neural plasticity and neuron‐glia interaction have been linked to the spinal machinery underlying the development of neuropathic pain.^43^, ^44^, ^45^ Thus, the cell type in which the proposed machinery occurs is very important. Our recent study has demonstrated that the expression of Cx43 is limited in astrocytes, but not neurons or microglia.^7^ However, this does not rule out the potential involvement of neurons and microglia, which will be further investigated in our future studies. And we did not test PKA activity in the present study because we have demonstrated that PDE4 inhibitors such as rolipram increase the activity and expression of PKA in the brain of mice.^46^, ^47^ There are accumulating studies showing the regulatory role of PKA in TNF‐α. Increased PKA produces a reduction of TNF‐α expression in astrocytes and other cells in vitro and/or in vivo,^48^, ^49^, ^50^, ^51^ supporting that TNF‐α is a downstream target of cAMP‐PKA signaling, although it is not clear whether PKA directly or indirectly regulates TNF‐α.

**FIGURE 7:**
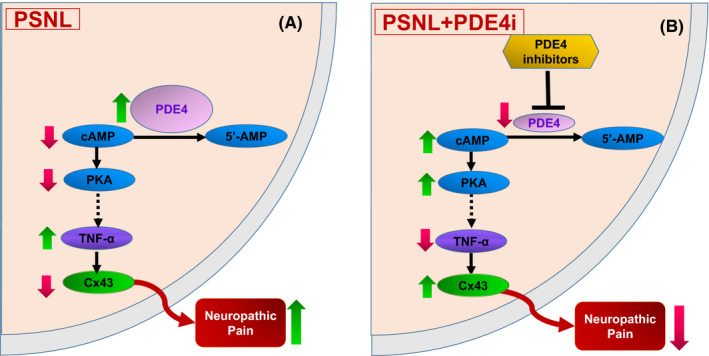
Schematic of the mechanisms by which PDE4 inhibitors attenuate neuropathic pain in PSNL mice. (A) In the spinal dorsal horn in PSNL mice, expression of PDE4 is induced, which causes decreases in cAMP levels and PKA activity, leading to activation of proinflammatory cytokines such as TNF‐α and downregulation of Cx43, eventually resulting in neuropathic pain. (B) Following treatment with PDE4 inhibitors (PDE4i) in PSNL mice, cAMP‐PKA signaling is activated and cytokines such as TNF‐α are suppressed, leading to increases in Cx43 and eventual suppression of neuropathic pain. PSNL, partial sciatic nerve ligation; PDE4, phosphodiesterase‐4; 5′‐AMP, 5′‐adenosine monophosphate; cAMP, cyclic AMP; PKA, protein kinase A; TNF‐α, tumor necrosis factor‐α; Cx43, connexin43

In conclusion, we demonstrated a distinct mechanism of neuropathic pain that is regulated by PDE4‐mediated intracellular signaling. PSNL induces expression of PDE4, most likely PDE4B, leading to decreases in cAMP levels and PKA activity. This causes increases in proinflammatory cytokines such as TNF‐α, and downregulation of Cx43 in the spinal dorsal horn, eventually resulting in neuropathic pain (Figure 7A). By contrast, inhibition of PDE4, in particular PDE4B, activates cAMP‐PKA signaling and suppresses TNF‐α, leading to increases in Cx43 in the spinal dorsal horn and eventual suppression of neuropathic pain (Figure 7B). To the best of our knowledge, this is the first demonstration of the role of Cx43 in PDE4‐mediated cAMP signaling in the regulation of neuropathic pain. The study provides valued clues on the mechanism whereby PDE4 inhibitors produce antinociceptive activity and will aid in the development of novel antinociceptive agents.

## CONFLICT OF INTEREST

The authors declare that they have no conflicts of interest.

## Supporting information

Fig S1. The original images of Figure 1B–E. Each group of mice were numbered from 1 to 10; red is sham group (sham1‐10); blue is PSNL group (PSNL1‐10)
Fig S2. The original images of Figure 5A,C. Each group of mice were numbered from 1 to 9; red is sham group (sh1‐9); blue is PSNL group (PS1‐9); green is rolipram treated PSNL group (Rol1‐9); vermilion is roflumilast treated PSNL group (Rof1‐8); black is CBX treated PSNL group (car1‐8)
Fig S3. The original images of Figure 5E,G. Each group of mice were numbered from 1 to 6; red is sham or vehicle group (sh1‐6 or veh1‐6); blue is PSNL group (PS1‐6); black is 24 h after TNF treated group (T24 1‐6); green is 48 h after TNF treated group (T48 1‐6); pink is etanercept treated PSNL group (eta1‐6)
Fig S4. The original images of Figure 6A–D. Each group of mice were numbered from 1 to 6; red is sham group (sh1‐6); blue is PSNL group (PS1‐6); green is rolipram treated PSNL group (Rol1‐6); vermilion is roflumilast treated PSNL group (Rof1‐6)

## Data Availability

The data that support the findings of this study are openly available in [repository name e.g “figshare”] at http://doi.org/[doi], reference number [reference number].
