# Supplementary material for: Inhibition of phosphodiesterase‐4 in the spinal dorsal horn ameliorates neuropathic pain via cAMP‐cytokine‐Cx43 signaling in mice
Source: CNS Neurosci Ther. 2022 Feb 14;28(5):749–60. doi: 10.1111/cns.13807 (PMC8981432; doi:10.1111/cns.13807)
Supplement: Supplementary file 1 — Fig S1. The original images of Figure 1B–E. Each group of mice were numbered from 1 to 10; red is sham group (sham1‐10); blue is PSNL group (PSNL1‐10) Fig S2. The original images of Figure 5A,C. Each group of mice were numbered from 1 to 9; red is sham group (sh1‐9); blue is PSNL group (PS1‐9); green is rolipram treated PSNL group (Rol1‐9); vermilion is roflumilast treated PSNL group (Rof1‐8); black is CBX treated PSNL group (car1‐8) Fig S3. The original images of Figure 5E,G. Each group of mice were numbered from 1 to 6; red is sham or vehicle group (sh1‐6 or veh1‐6); blue is PSNL group (PS1‐6); black is 24 h after TNF treated group (T24 1‐6); green is 48 h after TNF treated group (T48 1‐6); pink is etanercept treated PSNL group (eta1‐6) Fig S4. The original images of Figure 6A–D. Each group of mice were numbered from 1 to 6; red is sham group (sh1‐6); blue is PSNL group (PS1‐6); green is rolipram treated PSNL group (Rol1‐6); vermilion is roflumilast treated PSNL group (Rof1‐6) [file CNS-28-749-s001.pdf]

Supplemental Files： The original images (Fig.1B-3, Fig.5A,C,E,G, and Fig.6A-D) of western blot were submitted in the supplemental files.

Supplemental Fig.1

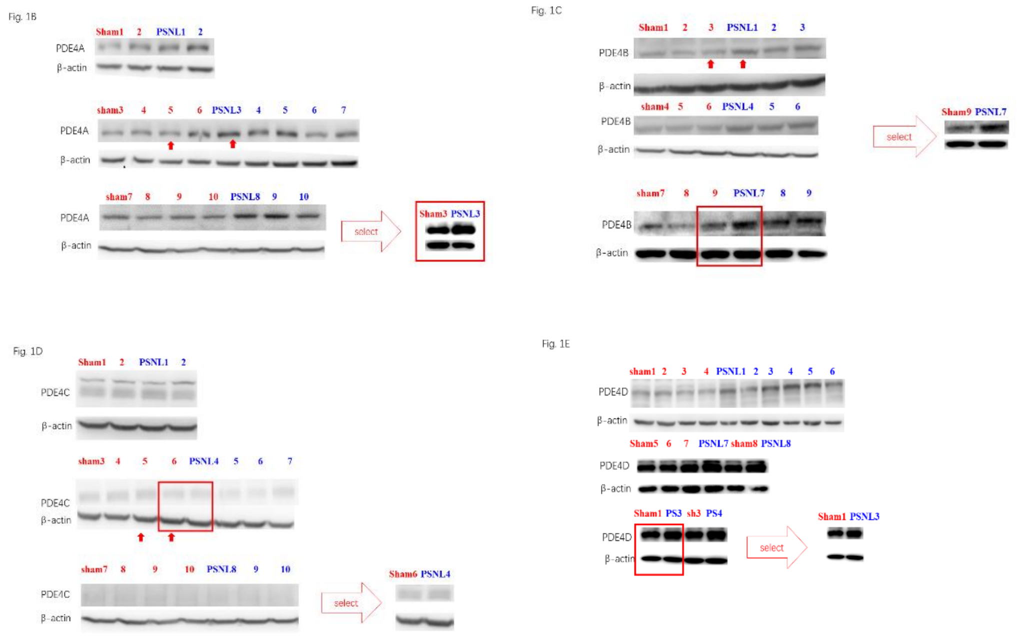

Supp Fig.1. The original images of Fig.1B-E. Each group of mice were numbered from 1 to 10, red is sham group (sham1-10); blue is PSNL group (PSNL1-10).

Supplemental Fig.2

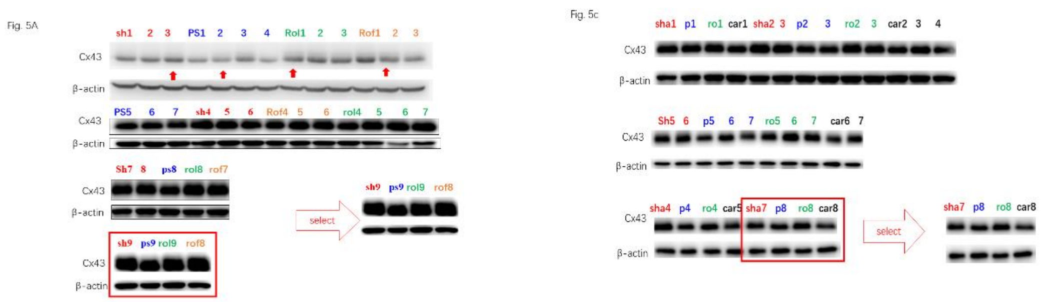

Supp Fig.2. The original images of Fig.5A and C. Each group of mice were numbered from 1 to 9, red is sham group (sh1-9); blue is PSNL group (PS1-9); green is rolipram treated PSNL group (Rol1-9); vermilion is roflumilast treated PSNL group (Rof1-8); black is CBX treated PSNL group (car1-8).

Supplemental Fig.3

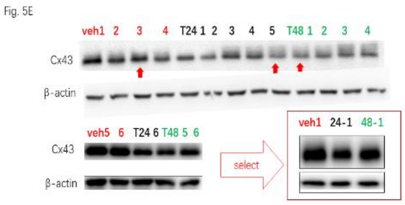

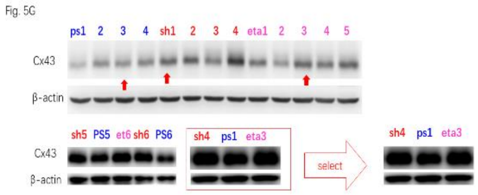

Supp Fig.3. The original images of Fig.5E and G. Each group of mice were numbered from 1 to 6, red is sham or vehicle group (sh1-6 or veh1-6); blue is PSNL group (PS1-6); black is 24 h after TNF treated group (T24 1-6); green is 48 h after TNF treated group (T48 1-6); pink is etanercept treated PSNL group (eta1-6).

Supplemental Fig.4

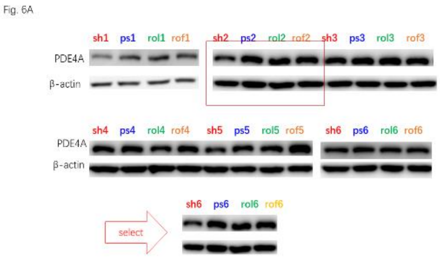

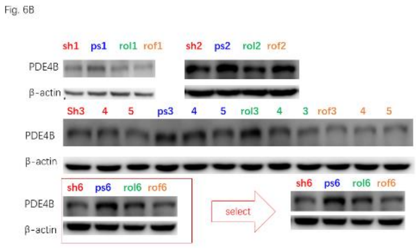

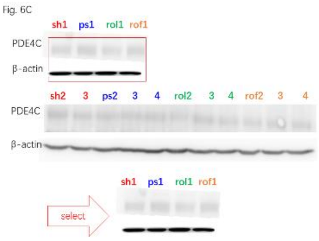

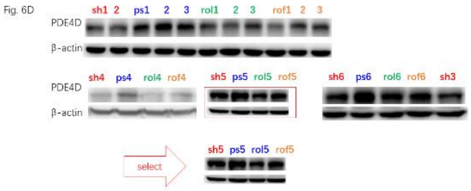

Supp Fig.4. The original images of Fig.6A-D. Each group of mice were numbered from 1 to 6, red is sham group (sh1-6); blue is PSNL group (PS1-6); green is rolipram treated PSNL group (Rol1-6); vermilion is roflumilast treated PSNL group (Rof1-6).
